# Chemical Analysis and Biological Activities of Extracts Isolated from Symbiotic *L. japonicus* Plants

**DOI:** 10.3390/life14020189

**Published:** 2024-01-27

**Authors:** Foteini D. Kalousi, Michail Tsakos, Christina N. Nikolaou, Achilleas Georgantopoulos, Anna-Maria G. Psarra, Daniela Tsikou

**Affiliations:** 1 Department of Biochemistry and Biotechnology, University of Thessaly, Biopolis, 41500 Larissa, Greece; 2 Department of Chemistry, National and Kapodistrian University of Athens, Panepistimiopolis, 15771 Athens, Greece; 3 Department of Natural Resources and Agricultural Engineering, Agricultural University of Athens, 75 Iera Odos Str., 11855 Athens, Greece

**Keywords:** plant extracts, arbuscular mycorrhizal fungi, *Rhizobium*, symbiosis, *Lotus japonicus*, anti-inflammatory activity, apoptosis, cell viability

## Abstract

Plants produce a wide variety of secondary metabolites, including compounds with biological activities that could be used for the treatment of human diseases. In the present study, we examined the putative production of bioactive molecules in the legume plant *Lotus japonicus,* which engages into symbiotic relationships with beneficial soil microorganisms. To monitor the production of secondary metabolites when the plant develops beneficial symbiotic relationships, we performed single and double inoculations with arbuscular mycorrhizal fungi (AMF) and nitrogen-fixing *Rhizobium* bacteria. Plant extracts from non-inoculated and inoculated plants were chemically characterized and tested for anti-proliferative, apoptotic, and anti-inflammatory effects on human HEK-293 cells. Both shoot and root extracts from non-inoculated and inoculated plants significantly reduced the HEK-293 cell viability; however, a stronger effect was observed when the root extracts were tested. Shoot and root extracts from *Rhizobium*-inoculated plants and shoot extracts from AMF-inoculated plants showed apoptotic effects on human cells. Moreover, both shoot and root extracts from AMF-inoculated plants significantly reduced TNFα-induced NF-κB transcriptional activity, denoting anti-inflammatory activity. These results suggest that symbiotic *L. japonicus* plants are enriched with metabolites that have interesting biological activities and could be further explored for putative future use in the pharmaceutical sector.

## 1. Introduction

Plant secondary metabolites (also called specialized metabolites or natural products), such as phenolics, terpenes, and nitrogen-containing compounds, are considered components of the survival strategy of plants and, in addition to other functions, help the plant cope with abiotic and biotic stresses and communicate with the environment [1,2,3]. A huge number of studies over the past years have highlighted the importance of many specialized metabolites, not only in plant, but also human health, as interesting biological activities have been attributed to these compounds. Nowadays, research focuses on the improvement of strategies to enhance the accumulation of such compounds in plant tissues in order to produce useful pharmaceuticals [4].

Engagement into symbiosis with beneficial soil microorganisms is a significant cause of changes in the plant metabolism. Most land plants (almost 80% of plant species) associate with arbuscular mycorrhiza fungi (AMF), which provides the plant with mineral elements (mainly phosphorus and nitrogen), improves water absorption, and enhances the plant tolerance to biotic and abiotic stresses [5,6]. In the last decade, a number of studies have reported the positive impact of plant inoculation with AMF on the production of important active compounds in the plant tissues [7,8]. Although the physical interactions between the microsymbionts and the plant are restricted to the roots, changes in the plant metabolism are observed in both the above and below ground tissues [9]. For example, the content of artemisinin, a compound that is used in malaria treatment, was significantly increased in the leaves of *Artemisia annua* plants after root inoculation with AMF [10,11,12]. Similarly, AMF inoculation improved the growth of the medicinal plant *Coleus forskohlii* and increased the forskolin (a compound used for treatment of heart disorders, cancer, etc.) content of tubers [13,14].

Legume plants have the capability to associate not only with AMF, but also with the nitrogen-fixing *Rhizobium* bacteria, which provide the plant with the often-limited macronutrient nitrogen. The importance of symbiotic nitrogen fixation in agriculture, together with the nutritional value of legumes in the human diet, have led to the extensive study of legumes, including research in the field of metabolomics [15]. Secondary metabolites (phenolic compounds, isoflavones, and saponins) from legumes, as well as proteins (like lectins), were suggested to have biological properties useful for the prevention and control of human diseases such as cancer, diabetes, inflammation, and cardiovascular diseases [16,17,18].

*Lotus japonicus* is a leguminous model plant which is mostly used for studies on symbiotic plant–microbe interactions. Metabolomics data are available for this plant, including changes in its primary and secondary metabolite content during symbiosis with *Rhizobium* [19]. *L. japonicus* has many traits that make it an excellent genetic model system (small genome, self-compatibility, and ease of transformation), and moreover, a large variety of resources (like transcriptomics data and mutant collections) are available, enabling the identification of the biosynthetic pathways responsible for the production of valuable bioactive molecules, like the antihistaminics rhodiocyanoside A and D [20,21].

In the present study, shoot and root ethyl acetate extracts were isolated from *L. japonicus* plants after single or double inoculation with *Rhizobium* bacteria and AMF. Plant extracts were analyzed for the determination of their chemical composition and were tested by biological assays, aiming at the identification of bioactive molecules with anti-proliferative, anti-inflammatory, and apoptotic properties.

## 2. Materials and Methods

### 2.1. Biological Material, Microbial Inoculation, and Growth Conditions

The plant used in this study was the *Lotus japonicus* L. ecotype Gifu B-129 wild type. The seeds were kindly provided by Prof. Jens Stougaard (Aarhus University, Aarhus, Denmark). *L. japonicus* seeds were surface scarified for 25 min with H_2_SO_4_, sterilized for 10 min in a solution containing 2% NaOCl, and kept in water for 12 h at 4 °C, then germinated on sterile wet filter paper for 10 days at 21 °C in a growth chamber (16 h light, 8 h dark). The plant seedlings were transferred to pots containing a 2:1 *v/v* mixture of sterile sand and vermiculite.

For inoculation with AMF, the inoculum (containing spores, hyphae, and small root pieces) was applied directly to each plant root. The seedlings were inoculated with (a) the commercially available *Rhizophagus irregularis* strain DAOM (Agronutrition) by applying almost 100 spores per root or (b) a *Rhizophagus irregularis* strain isolated from a certified organic farm in Greece [22] by applying almost 15 spores per root. The plants were watered once per week, alternately with deionized H_2_O or a modified Long Ashton nutrient solution (SLA) [23].

For inoculation with *Rhizobium*, *Mesorhizobium loti* cv. R7A liquid cultures were grown at 28 °C in yeast mannitol broth for two days and diluted to an optical density of 0.02 at λ = 600. One ml of bacterial suspension was applied directly to each root. The plants were watered three times per week, alternately with deionized H_2_O or nitrogen-free Hoagland’s nutrient solution [24].

For the double inoculation, the rhizobial liquid culture was mixed with the fungal inoculum and the mixture was applied to each root. The plants were watered once per week, alternately with deionized H_2_O or SLA.

All plants were grown for five weeks in a controlled environment, in growth chambers with a 16 h day and 8 h night cycle. AMF single-inoculated plants and double-inoculated plants were grown at 24–25 °C, and *Rhizobium* single-inoculated plants were grown at 21 °C. All plants were harvested five weeks post inoculation with the microbes.

### 2.2. Testing Root Colonization by Microsymbionts

For the quantification of mycorrhizal colonization, four or five (out of forty) randomly selected AMF-inoculated roots were stained with 5% ink in 5% acetic acid solution [25]. The (%) percentage of mycorrhizal colonization was estimated by microscopic examination of slides containing almost 15 cm of stained root. Extracts were isolated from plants that had been colonized by the fungi at a rate of 35–55%. Similarly, successful root colonization by *Rhizobium* bacteria was assessed by observing nodules under a stereoscope.

### 2.3. Treatments, Tissue Harvesting, and Extraction

Extracts were isolated from plants which had undergone the following treatments:

- Non-inoculated plants grown at 21 °C (control sample “C1”)
- Non-inoculated plants grown at 24–25 °C (control sample “C2”)
- Plants inoculated with the *Rhizobium* bacterium *M. loti* (“R” sample)
- Plants inoculated with the Greece-isolated *R. irregularis* strain (“AMF” sample)
- Plants inoculated with the *R. irregularis* DAOM strain (“AMF-D” sample)
- Plants double-inoculated with *Rhizobium* and AMF (DAOM) (“R + D” sample)

Plants were separated into above-ground and below-ground tissues, that we named “shoots” and “roots”, respectively. Almost 35 plants were harvested for the production of a single shoot or root sample. The tissues were collected in a 50 mL falcon tube and immediately frozen in liquid nitrogen. The samples were kept at −80 °C until lyophilized. Then, tissues were ground with a mortar and pestle and extracted with 20 mL of ethyl acetate. The extract was passed through a filter paper and collected in a glass rounded flask. The organic solvent was evaporated, and the samples were dried by nitrogen gas. Dried samples were resuspended in dimethyl sulfoxide (DMSO) at a concentration of 10 mg/mL.

### 2.4. Chemical Analysis of the Extracts

The methodology followed for the chemical analysis of the *L. japonicus* ethyl acetate extracts is described in detail in the Supplementary Materials.

### 2.5. Cell Culture

Human embryonic kidney HEK-293 cells were obtained from the American type culture collection (ATTC) and were cultured at 37 °C and 5% CO_2_ humidity in DMEM, supplemented with 10% FBS, 2 mM L-glutamine, and 100 units/mL penicillin/streptomycin (pen/strep). HEK-293 cells exhibit high efficiency in transfection experiments.

### 2.6. Cell Viability Assay

The effect of the isolated extracts on the cell viability of HEK-293 cells was assessed by applying an MTT assay as previously described [26]. Briefly, HEK-293 cells were plated in a 96-well plate, at a density of 1.5 × 10^4^ cells/well for 24 h in DMEM, supplemented with 10% FBS, 2 mM L-glutamine, and 100 units/mL pen/strep. Then, cells were incubated with extracts from roots and shoots from plants single- or double-inoculated with *Rhizobium* and AMF or from non-inoculated plants for 48 h. Then, 0.5 mg/mL MTT reagent was added for 3–4 h. Subsequently, the produced formazan crystals were dissolved in 100% isopropanol via shaking. Finally, the absorbance was measured at 570 nm, using a multimode plate reader (EnSpire, PerkinElmer, Beaconsfield, UK). Background absorbance was also measured at 690 nm, as a reference.

### 2.7. Anti-Inflammatory Activity Assessment

To assess the potential anti-inflammatory activity of the isolated extracts, NF-κB transcriptional activity was measured by applying a luciferase reporter gene assay as previously described [27]. Concisely, HEK-293 cells were plated on 24-well plates (3 × 10^4^ cells/well) in DMEM medium supplemented with 10% FBS, 2 mM L-glutamine, and 100 units/mL pen/strep. After 24 h, cells were transiently co-transfected using calcium phosphate with a NF-κΒ response element (NF-κB-RE) promoter-driven luciferase construct and a β-galactosidase reporter construct. At 14–16 h post transfection, cells were washed in fresh medium for 24 h and then treated with 20 ng/mL TNFα diluted in ddH_2_O in the presence or absence of the indicated amounts of the isolated extracts or 1 μΜ Dexamethasone (DEX) (diluted in EtOH) for 6 h. DEX-treated cells were used as the positive control since DEX, the synthetic glucocorticoid, is well-known for its anti-inflammatory activity [28]. DMSO/EtOH-treated cells were used as the negative control. Then, cells were lysed in reporter lysis buffer and the enzymatic activities of luciferase and β-galactosidase were measured using a chemiluminometer (LB 9508; Berthhold, www.berthhold.com, accessed on 5 August 2014). β-galactosidase activity measurement was used for normalization of luciferase activity (RLU).

### 2.8. Western Blot Analysis

HEK-293 cells were plated on 6-well plates (2 × 10^5^ cells/well) and were grown in DMEM medium supplemented with 10% FBS, 2 mM L-glutamine, and 100 units/mL pen/strep for 48 h. Then, cells were treated with the indicated amounts of the isolated extracts for an additional 48 h. DMSO treatment at the respective dilution (1/500 or 1/200) was used as the control condition. Cells were then washed in PBSx1 and lysed in Lysis buffer (20 mM Tris pH: 7.5, 250 mM NaCl, 0.5% Triton, 3 mM EDTA) supplemented with cocktail protease inhibitors, 2 mM DTT, and 0.1 mM PMSF. Protein concentration assessment was followed applying a Bradford assay. Cell extracts were electrophoresed in discontinuous SDS-PAGE and Western blotted. Procaspase-3, procaspase-9, and β-actin protein levels were evaluated by using specific antibodies as previously described [27]. β-actin protein levels were assessed for the normalization of the results. Enhanced chemiluminescence was applied for protein bands’ detection e.

### 2.9. Statistical Analysis

All results are expressed as mean ± SD. Data were analyzed by a one-way analysis of variance (ANOVA) or a two-way ANOVA followed by Tukey’s post-hoc test using SPSS (IBM SPSS Statistics 23) or Stat Plus (StatPlus LE v7) software, respectively. Differences were considered significant at a two-tailed *p* value < 0.05.

## 3. Results

### 3.1. Lotus Japonicus Metabolite Profiling under Different Microbial Inoculation Treatments

Ethyl acetate extracts from non-symbiotic and symbiotic *L. japonicus* plants were analyzed by LC-QTOF-MS/MS for the identification of their chemical composition. The analysis included both shoot and root extracts, isolated from non-inoculated plants (control), plants single-inoculated with the *Rhizobium* bacterium *M. loti* (R) or two strains of the AMF *R. irregularis* (AMF, AMF-D), or plants double-inoculated with *M. loti* and *R. irregularis* (R + D). The extracts contained both primary and secondary metabolites; however, this study focused on the identification and quantification of secondary metabolites and fatty acids, aiming at the investigation of bioactive compounds. The analysis identified a total of 65 secondary metabolites, most of which belong in the general category of phenols, and a few molecules belonging to terpenes and N-containing compounds (Table S1). Selected compounds identified in the shoot and root extracts were semi-quantified (Table S2) and the results are presented in Table 1 as ratios of the quantities found in inoculated vs. non-inoculated (control) plants.

The chemical analysis showed huge changes in the accumulation of many metabolites after the inoculation of plants with any of the microsymbionts compared to the control (non-inoculation) condition. Most metabolites were affected by at least one of the microbial inoculation treatments, and only three (chalconaringenin, vestitone, and coniferyl acetate) out of the 52 metabolites semi-quantified were not affected by any of the treatments. Of the 49 metabolites that were affected by the microbial inoculation, 43 metabolites were affected by AMF inoculation and 41 by *Rhizobium* inoculation. In roots, inoculation with AMF, AMF-D, R, and R + D resulted in a remarkable change (five-fold or greater change) of twelve, twenty, ten and sixteen metabolites, respectively. As expected, changes were observed not only in roots, where microbial colonization takes place, but also in shoots. Thus, similarly, in shoots, inoculation with AMF, AMF-D, R, and R + D resulted in a remarkable change (five-fold or greater change) of fourteen, five, twelve, and six metabolites, respectively. Almost all of the identified metabolites were detected in all samples analyzed, except for quercetin-3-rutinoside, which was shoot-specific, and eriodictyol, which was *Rhizobium*-specific (Table 1).

Inoculation with *Rhizobium* and AMF did not cause similar metabolic changes in the tissues and resulted in different metabolic patterns, as many metabolites that were induced by one microsymbiont were reduced by the other. For example, in root extracts, accumulation of several metabolites, like hydroxycaffeic acid, vestitol, lotaustralin, phenylalcetaldehyde, and caffeic acid hexoside, was reduced by AMF inoculation and induced by *Rhizobium* inoculation. Considering the two *R. irregularis* strains (AMF and AMF-D) tested, comparison between the strains showed that differences are observed in the accumulation levels of the metabolites; however, both strains caused plant metabolic changes towards the same direction (induction or reduction), resulting in a similar metabolic profile. It is noteworthy that inoculation with any of the AMF strains caused a reduction in the accumulation levels of the metabolites. Specifically, in roots, the levels of twenty-two metabolites were reduced after inoculation with AMF, and no metabolite was induced, compared to control non-inoculated plants; similarly, in shoots, the levels of twenty-six metabolites were reduced and only three metabolites were induced. Contrastingly to AMF inoculation, *Rhizobium* inoculation resulted in elevated levels of many metabolites. More specifically, in *Rhizobium*-inoculated roots, the levels of twenty-one metabolites were increased and the levels of nine metabolites were reduced; similarly, in *Rhizobium*-inoculated shoots, the corresponding numbers were twenty and twelve. Considering the double-inoculated plants, the levels of twenty-nine metabolites were increased and only three metabolites were reduced in roots, a metabolic profile that resembles that of *Rhizobium*-inoculated roots. However, in shoots, the levels of four metabolites were increased and nine metabolites were reduced, which resembles the AMF-inoculation metabolic profile (Table 1).

Some metabolites mostly accumulated in shoot tissues, whereas some others were more prominent in roots (Table 2). A comparison between the metabolite accumulation in the root and shoot extracts of non-inoculated (control) plants showed that out of fifty-four metabolites analyzed, six metabolites were more (two-fold or greater) accumulated in roots than shoots, and twenty-three metabolites were more (two-fold or greater) accumulated in shoots than roots. Inoculation with the microsymbionts caused changes in the metabolite allocation between shoot and roots; however, for most of the metabolites, the changes observed were towards the same direction (higher accumulation in roots or shoots), resulting in a similar metabolite root to shoot accumulation pattern. Thus, in general, most of the metabolites analyzed were found in higher abundance in shoots rather than roots, regardless of the plant symbiotic or non-symbiotic status. Despite this general observation, it is noteworthy that specific inoculation treatments resulted in remarkable changes in the accumulation of specific metabolites. For example, the accumulation of (6aR,11aR)-3,9-Dihydroxypterocarpan, which was seen equally distributed between shoots and roots in non-inoculated (control) plants, was 10 times up in roots compared to shoots of *Rhizobium*-inoculated plants, as well as *Rhizobium*–AMF double-inoculated plants, an effect that was not observed by AMF single inoculation. Another example is genistin, which was accumulated in shoots rather than roots; however, this pattern changed after single inoculation with *Rhizobium,* resulting in a remarkably high accumulation in root extracts. Similarly, double inoculation with AMF and *Rhizobium* resulted in a remarkably high accumulation of maachiain in roots, an effect that was not seen in the other treatments (Table 2).

### 3.2. Effects of L. japonicus Extracts on HEK-293 Cell Viability

**Figure 1:**
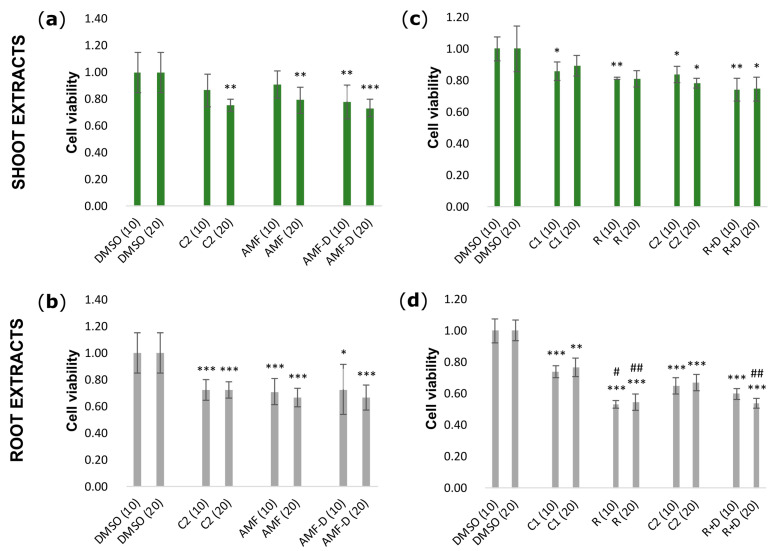
Effects of extracts of non-symbiotic and symbiotic *L. japonicus* plants on HEK-293 cell viability. Cytotoxicity was evaluated by an MTT assay, following the application of shoot (**a**,**c**) or root (**b**,**d**) extracts to HEK-293 cell cultures at a concentration of 10 μg/mL (10) or 20 μg/mL (20) and 48 h incubation. The viability of control DMSO-treated cells was considered 1 and all measurements were normalized accordingly. DMSO: control DMSO-only treatment; C1: extracts from non-inoculated plants grown at 21 °C; C2: extracts from non-inoculated plants grown at 24–25 °C; AMF: extracts from AMF-inoculated plants (Greek isolate); AMF-D: extracts from AMF-inoculated plants (DAOM strain); R: extracts from *Rhizobium*-inoculated plants; R + D: extracts from *Rhizobium*–AMF double-inoculated plants. Bars show means and standard deviation (*n* = 4–6). Comparisons are between samples of the same concentration. One-way ANOVA: * *p* < 0.05, ** *p* < 0.01, *** *p* < 0.001 (comparison with DMSO treatment) and ^#^ *p* < 0.05, ^##^ *p* < 0.01 (comparison between inoculated and non-inoculated plants).

To evaluate the putative effects of *L. japonicus* shoot and root ethyl acetate extracts on HEK-293 cell viability, an MTT assay was implemented. Extracts were applied to HEK-293 cells at a concentration of 10 μg/mL or 20 μg/mL, and cell viability was estimated 48 h later. Shoot extracts from AMF-inoculated plants caused a slight reduction (almost 0.2-fold) in cell viability (Figure 1a), and root extracts of the same plants caused an almost 0.3-fold reduction in cell viability (Figure 1b), compared to that observed when the cells were treated with DMSO only. A similar slight reduction in cell viability, almost 0.2- and 0.25-fold (compared to DMSO treatment), was caused by shoot extracts from plants inoculated with *Rhizobium* or double-inoculated with *Rhizobium* and AMF, respectively (Figure 1c). However, a high reduction in cell viability was observed in cell cultures treated with root extracts from *Rhizobium* single-inoculated or *Rhizobium*–AMF double-inoculated plants. Specifically, the levels of HEK-293 cells’ viability were found to be almost 0.5-fold reduced by both treatments compared to those measured in DMSO-treated cells (Figure 1d). It is noteworthy that extracts from non-inoculated plants (samples C1 and C2) were also seen to reduce HEK-293 cells’ viability (Figure 1); however, considering the root samples, the effect was not as strong as that observed by the application of extracts from *Rhizobium*-inoculated plants (Figure 1d). Considering the comparisons between the two different concentrations of extracts tested in this assay, no significant differences were observed.

### 3.3. Apoptotic Activities of L. japonicus Extracts

**Figure 2:**
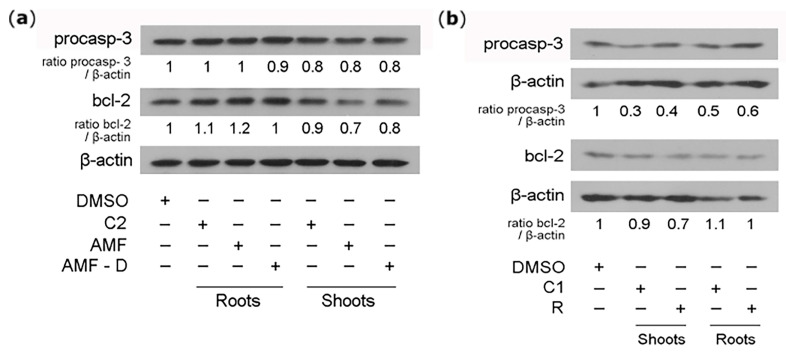
Apoptotic properties of extracts of non-symbiotic and symbiotic *L. japonicus* plants on HEK-293 cells. A Western blot analysis of procaspace-3 and bcl2 after the application of shoot and root extracts to human cell cultures at a concentration of 20 μg/mL and 48 h incubation. (**a**) Protein levels after the application of extracts from AMF-inoculated plants or non-inoculated plants grown at 24 °C, (**b**) protein levels after the application of extracts from *Rhizobium*-inoculated plants or non-inoculated plants grown at 21 °C. Data are expressed as ratios of the levels of the apoptosis-related proteins to the respective levels of β-actin. The relative band density of control DMSO-treated cells was set as 1. DMSO: control DMSO-only treatment; C1: extracts from non-inoculated plants grown at 21 °C; C2: extracts from non-inoculated plants grown at 24–25 °C; AMF: extracts from AMF-inoculated plants (Greek isolate); AMF-D: extracts from AMF-inoculated plants (DAOM strain); R: extracts from *Rhizobium*-inoculated plants.

To assess putative pro-apoptotic and apoptotic properties of the plant extracts, a Western blot analysis of procaspace-3 and bcl2 protein levels was performed after the application of shoot and root ethyl acetate extracts to HEK-293 cells. Extracts from non-inoculated, AMF-inoculated, and *Rhizobium*-inoculated *L. japonicus* plants were applied to HEK-293 cell cultures at a concentration of 20 μg/mL, and the human cells were incubated for 48 h in a hormone-free medium. The treatment of HEK-293 cells with shoot extracts from non-inoculated (C2) or AMF-inoculated plants (grown at 24–25 °C) resulted in a slight reduction in procaspace-3 and bcl2 protein levels (approximately 0.2-fold), and no effect was observed when root extracts from the same plants were tested (Figure 2a). A significant decrease in procaspace-3 protein levels was observed in HEK-293 cells treated with either shoot extracts (more than 0.5-fold) or root extracts (approximately 0.5-fold) from *Rhizobium*-inoculated plants and non-inoculated (C1) plants grown at 21 °C (Figure 2b). Moreover, the levels of bcl2 protein were affected by the application of shoot extracts (approximately 0.3-fold), but not root extracts, from *Rhizobium*-inoculated plants (Figure 2b).

### 3.4. Anti-Inflammatory Properties of L. japonicus Extracts

**Figure 3:**
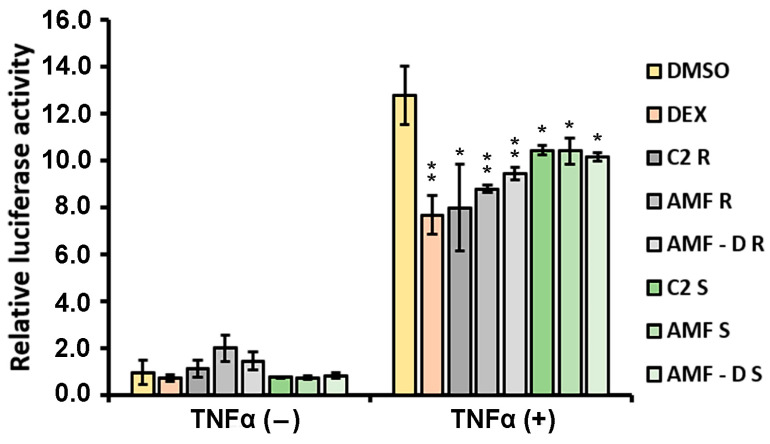
The anti-inflammatory activities of extracts from AMF-inoculated plants. The anti-inflammatory activities of the non-inoculated and AMF- and AMF-D-inoculated plant extracts were evaluated by assessment of the suppression of TNFα-induced NF-κB transcriptional activation in HEK-293 cells. HEK-293 cells were co-transfected with the NF-κB-luciferase reporter and the β-galactosidase reporter constructs and, subsequently, were treated with 20 μg/mL plant extract or with 1 μM DEX for 6 h in the presence or absence of 20 ng/mL TNFα in a hormone-free medium. The results are expressed as relative luciferase activity normalized against β-galactosidase activity. The relative luciferase activity in control TNFα-minus DMSO-treated cells was set at 1. DMSO: control DMSO-only treatment; C2: extracts from non-inoculated plants grown at 24–25 °C; AMF: extracts from AMF-inoculated plants (Greek isolate); AMF-D: extracts from AMF-inoculated plants (DAOM strain); R: root extract; S: shoot extract. Bars show means and SD (*n* = 3). Two-way ANOVA: * *p* < 0.05, ** *p* < 0.01 (comparison with TNFα-plus DMSO treatment).

The putative anti-inflammatory activities of the plant extracts were evaluated by an NF-κB-associated luciferase/β-galactosidase reporter gene assay. Transfected HEK-293 cells were treated with 20 μg/mL plant extract or with 1 μM DEX for 6 h in the presence or absence of TNFα. The treatment of HEK-293 cells with either shoot or root extracts from non-inoculated (C2) or AMF-inoculated plants (grown at 24–25 °C) resulted in statistically significant inhibition of TNFα-induced NF-κB transcriptional activation (Figure 3). Specifically, TNFα-treated cells induced greater NF-κΒ transcriptional activation up to 13-fold, while DEX caused statistically significant reduction up to 40%, as expected. Treatment with root extracts from C2, AMF, and AMF-D plants reduced TNFα-induced NF-κΒ transcriptional activation up to 35, 30, and 25%, respectively. Shoot extracts from the same plants also reduced TNFα-induced NF-κΒ transcriptional activation, but to a lesser extent (18–20%). No statistically significant difference was observed between extracts from AMF- and AMF-D-inoculated plants and extracts from control non-inoculated plants. No anti-inflammatory activity was observed after the application of extracts from *Rhizobium*-inoculated plants to HEK-293 cells, even when a higher concentration (40 μg/mL) of extracts was tested (Supplementary Figure S1).

## 4. Discussion

In the present study, we took advantage of the wide variety of secondary metabolites produced in plants, and we triggered the enhancement of the variation of the *L. japonicus* plant metabolic profile by inoculating the plant roots with beneficial soil microorganisms to investigate the putative accumulation of bioactive compounds in plant tissue extracts. Plant single inoculations with *M. loti* (the *L. japonicus Rhizobium* microsymbiont) or two *R. irregularis* (AMF) strains and double inoculation with *Rhizobium* and AMF resulted in altered metabolic profiles of shoot and root ethyl acetate extracts (Table 1). Microbial inoculation affected not only the metabolite content within the shoots or roots, but also the metabolite distribution between the shoots and roots (Table 2).

The chemical analysis showed that the plant extracts were predominantly enriched in phenolic compounds, although a few metabolites belonging to terpenes and N-containing compounds were also identified (Table 1, Table 2, and Table S1). The most abundant group of metabolites identified in the extracts were flavonoids (48% of the total secondary metabolites identified) (Table S1), a large family of phenolic compounds that are ubiquitous in plants and play important roles in the symbiosis with rhizobia [29], as well as plant interactions with AMF [30,31]. The *L. japonicus* extracts were enriched in isoflavones (Table S1), a subclass of flavonoids which are characteristic molecules in leguminous plants, as they are most commonly found in the legume (Fabaceae) family [32]. Isoflavones are well-known for their biological activities, including estrogenic and anticancer activities, beneficial for human health. Clinical studies have claimed the benefits of specific isoflavones (like genistein and daidzein) against cancer, cardiovascular disease, osteoporosis, and postmenopausal symptoms [33]. Moreover, flavonoids are known as potent antioxidants and also exert anti-inflammatory properties by inhibiting regulatory enzymes or transcription factors involved in inflammation [34]. In addition to flavonoids, compounds with known biological activities, such as hydroxycaffeic acid, a phenolic acid with anticancer activity [35]; coniferaldehyde, a phenylpropanoid with anti-inflammatory activity [36]; lariciresinol, a lignan which induces apoptosis [37]; and compounds with multiple biological activities (anti-inflammatory, anticancer, antioxidant, and antibacterial) like the phenolic aromatic compound eugenol [38] and the triterpene acid ursolic acid [39], were also detected in the *L. japonicus* extracts.

Assessment of the biological activities of shoot and root extracts from non-inoculated or inoculated plants revealed the moderate apoptotic activities of extracts even from non-inoculated plants. Extracts from roots exhibited relatively increased anti-proliferative activity which was accompanied by induction of apoptosis, as indicated by the caspase-3 activation in HEK-293 cells treated with root extracts from non-inoculated or *Rhizobium*-inoculated plants grown at 21 °C. Induction of apoptosis was increased when extracts were derived from plants grown at 21 °C, an effect that was not observed with extracts of plants (inoculated or not) grown at 24–25 °C, which may reflect the effects of high temperature on different aspects of plant physiology and metabolism, given that the optimal growth temperature of *L. japonicus* is 21 °C. Shoot extract’s anti-proliferative activity was also accompanied by the induction of apoptosis in HEK-293 cells treated with extracts from either inoculated or non-inoculated plants, as indicated by the reduction in procaspase-3 and bcl2 protein levels. *Rhizobium* inoculation, in accordance with its suppressive effect on cell viability, strengthened the apoptotic properties of the root extracts, which may be associated with the observed *Rhizobium* inoculation-induced accumulation of plant metabolites known for their anticancer and/or apoptotic properties, such as hydroxycaffeic acid [35], lariciresinol [37], formononetin [40], portulacaxanthin [41], oleic acid, and glycerol tributanoate [42,43]. The less toxic extracts from the AMF- and AMF-D-inoculated plants were further assessed for their potential anti-inflammatory activity. A luciferase assay revealed that root and shoot extracts from non-inoculated and AMF- and AMF-D-inoculated plants showed suppressive effects on TNFα-induced NF-κΒ activation. Interestingly, root extracts exhibited inflammatory activity comparable to that of the synthetic glucocorticoid DEX, which is well-known for its anti-inflammatory activity, exerted among others via suppression of the transcriptional activation of the inflammatory factor NF-κΒ [44]. This action may be attributed to root extracts’ composition, which is enriched by metabolites known for their involvement in inflammation, such as vanillic acid [45] and gamma tocopherol [46]. Although the microbial inoculation did not enhance the anti-inflammatory properties of the plant tissues, the knowledge of their not reducing the anti-inflammatory potential of the plant extracts is also useful, because these microbes benefit the plant, and such symbiotic associations are desirable in crops. The finding that the extracts possess significant anti-inflammatory properties, while also possessing minor anti-proliferative properties (denoting that the extracts are not toxic), makes their further exploitation meaningful and promising for future pharmaceutical use.

## 5. Conclusions

Bioactivity assays performed with *L. japonicus* shoot and root ethyl acetate extracts uncovered their moderate anti-proliferative, apoptotic, and anti-inflammatory properties, suggesting the accumulation of bioactive molecules in the tissues of this plant. Engagement into symbiotic relationships with rhizobia and AMF altered the metabolic profile of the plant and influenced the biological activities of the plant extracts. Our results suggest that asymbiotic *L. japonicus* plants have the ability to produce bioactive molecules, and this trait is not only maintained, but also enhanced, upon inoculation with beneficial microbes. This indicates that the production of interesting molecules in *L. japonicus* tissues can be accomplished by using microbial fertilizers. This knowledge is valuable because it promises the successful production of interesting bioactive molecules in legume plants in a sustainable way, without the addition of high amounts of chemical fertilizers. Future work may focus on specific molecules that possess the desired biological activities and explore putative additional properties of these extracts, like antioxidant and antimicrobial activities.

## Figures and Tables

**Table 1:** Identified metabolites of *L. japonicus* ethyl acetate extracts. Data are presented as ratios to the corresponding control treatment (non-inoculated plants). In bold: two-fold or greater change; In red: five-fold or greater increase; In blue: five-fold or greater decrease.

Metabolites	Shoot Extracts					Root Extracts				
	* Control	AMF	AMF-D	R	R + D	Control	AMF	AMF-D	R	R + D
**Phenolics**										
vanillic acid	1	**0.4**	1.1	1.2	0.9	1	**0.5**	**0.2**	**3.0**	1.5
apigenin 7-glucoside	1	**0.4**	**0.3**	**5.4**	**3.4**	1	1.9	**2.1**	**0.4**	**2.0**
cyanidin3-rytinoside	1	**0.4**	**0.1**	**2.4**	1.7	1	1.3	0.7	0.6	**6.8**
hydroxycaffeic acid	1	0.6	1.1	**3.4**	1.7	1	**0.0**	**0.4**	**10.7**	**7.4**
isoorientin	1	0.6	**0.5**	**3.2**	0.8	1	0.9	0.8	1.2	0.9
isorhoifolin	1	**0.3**	**0.3**	**5.1**	**2.9**	1	**0.5**	**0.2**	**4.6**	**3.1**
lariciresinol	1	0.6	0.6	0.6	1.0	1	1.3	**2.5**	1.7	**5.3**
luteolin-5-glucoside	1	0.6	**0.5**	**3.2**	0.8	1	0.9	0.8	1.2	0.9
quercetin-3-rutinoside	1	**0.2**	**0.3**	**4.7**	1.3	N/A	N/A	N/A	N/A	N/A
gamma tocopherol	1	**0.0**	1.0	**7.2**	1.1	1	**0.1**	**0.2**	**2.3**	**2.3**
m-coumaric acid	1	0.6	**0.3**	1.8	1.0	1	0.9	0.9	**19.5**	**8.2**
maachiain	1	**0.1**	**0.3**	**0.4**	**0.4**	1	**0.2**	**0.2**	**0.5**	**11.3**
glycinol	1	1.3	1.2	1.2	1.2	1	1.0	0.9	1.1	1.0
phaseollin	1	0.6		**3.5**	1.8	1	**0.1**	**0.0**	**0.4**	**2.8**
vestitol	1	**0.0**	**0.4**	**0.1**	**0.2**	1	**0.1**	**0.1**	**16.8**	**4.5**
sophorol	1	**0.0**	0.6	**0.4**	1.5	1	**0.1**	**0.1**	**0.3**	**8.5**
(6aR,11aR)-3,9-Dihydroxypterocarpan	1	**0.2**	**0.3**	0.7	**0.2**	1	1.0	**0.2**	**6.6**	**2.4**
2′-Hydroxyformononetin	1	**0.1**	**0.3**	**0.4**	**0.4**	1	**0.2**	**0.2**	**0.5**	**11.3**
astragalin	1	0.6	**0.5**	**3.2**	0.8	1	0.9	0.8	1.2	0.9
chalconaringenin	1	1.3	1.2	1.2	1.2	1	1.0	0.9	1.1	1.0
delphinidin	1	**0.1**	**0.1**	**0.1**	**0.1**	1	**0.3**	**0.1**	**0.1**	**0.0**
eugenol	1	**60.9**	**72.2**	**80.8**	**79.1**	1	1.0	1.2	**2.2**	1.6
formononetin	1	**0.0**	**0.4**	**0.2**	**0.3**	1	**0.3**	**0.0**	**2.8**	1.6
genistein	1	**0.2**	**0.1**	**0.1**	**0.1**	1	**0.4**	**0.2**	0.6	**0.4**
genistin	1	0.7	**0.5**	**0.0**	**2.1**	1	1.9	**2.1**	1.7	**2.0**
homogentistic acid	1	**0.4**	1.1	1.2	0.9	1	**0.5**	**0.2**	**3.0**	1.5
leucopelargonidin	1	**5.2**	**4.6**	**4.4**	**4.3**	1	1.0	1.1	1.0	1.0
lotaustralin	1	**0.3**	0.7	1.0	1.8	1	**0.4**	**0.1**	**8.0**	**9.5**
pelargonidin	1	**0.2**	**0.1**	**0.1**	**0.1**	1	**0.4**	**0.2**	0.6	**0.0**
phenylalcetaldehyde	1	0.6	0.6	**6.2**	1.3	1	**0.1**	**0.0**	**12.6**	**5.8**
sinapaldehyde	1	0.8	0.9	**0.4**	0.8	1	1.0	1.0	1.0	0.6
vestitone	1	0.8	0.7	0.6	0.7	1	0.9	0.8	1.0	0.9
coniferyl acetate	1	1.2	1.1	1.2	1.1	1	1.0	1.0	1.1	1.1
astragalin	1	0.6	**0.5**	**3.2**	0.8	1	0.9	0.8	1.2	0.9
caffeic acid hexoside	1	0.6	1.1	**3.4**	1.7	1	**0.0**	**0.4**	**10.7**	**7.4**
coniferaldehyde	1	**0.5**	**0.4**	**3.1**	1.6	1	0.9	**0.1**	**3.2**	**4.5**
diosmetin	1	**0.0**	0.6	**0.4**	1.5	1	**0.1**	**0.1**	**0.3**	**8.5**
eriodictyol	N/A	N/A	N/A	1	N/A	N/A	N/A	N/A	**5.3**	N/A
**N-containing compounts**										
caffeine	1	**0.5**	**0.4**	1.4	**2.2**	1	**0.3**	**0.3**	**2.1**	**3.5**
cinchonidine	1	1.1	1.0	1.2	1.0	1	1.1	**0.4**	**0.1**	1.1
theobromine	1	0.8	**0.1**	**0.2**	0.8	1	**0.2**	**0.3**	0.8	**2.2**
portulacaxanthin	1	**4.0**	**9.0**	**26.2**	**0.5**	1	0.6	**2.4**	**8.4**	1.2
**Terpenes**										
oleanolic acid	1	**0.3**	0.6	**2.4**	0.9	1	**0.1**	**0.0**	1.8	0.7
ursolic acid	1	**0.3**	0.6	**2.4**	0.9	1	**0.1**	**0.0**	1.8	0.7
menthone	1	**0.4**	0.7	**2.0**	1.1	1	0.7	0.8	0.9	0.8
**Sterols**										
desmosterol	1	1.2	1.3	0.7	0.6	1	1.2	1.0	0.6	**0.5**
**Fatty acids**										
myristoleic	1	**0.4**	**0.5**	0.6	1.8	1	0.7	0.7	1.3	**8.0**
octadecanoic acid	1	0.6	0.8	0.7	0.7	1	1.1	0.8	0.8	1.8
oleic acid	1	0.6	**0.5**	0.8	0.6	1	0.7	1.1	**2.9**	**2.0**
palmitoleic acid	1	0.7	0.7	0.6	1.4	1	1.1	1.0	1.4	**6.9**
pentadecanoic acid	1	0.8	0.8	0.6	1.2	1	0.9	0.7	1.1	**2.8**
glycerol tributanoate	1	**0.5**	**0.5**	0.7	1.0	1	1.0	**2.6**	**2.3**	**5.9**

* Control: extracts from non-inoculated plants; AMF: extracts from AMF-inoculated plants (Greek isolate); AMF-D: extracts from AMF-inoculated plants (DAOM strain); R: extracts from *Rhizobium*-inoculated plants; R + D: extracts from *Rhizobium*–AMF double-inoculated plants.

**Table 2:** Accumulation of metabolites of *L. japonicus* ethyl acetate extracts in roots vs. shoots. Data are presented as ratios of metabolite accumulation in roots to metabolite accumulation in shoots. In bold: two-fold or greater change; In red: higher accumulation in roots than shoots (five-fold or greater); In blue: higher accumulation in shoots than roots (five-fold or greater).

Metabolites	Root/Shoot Ratios				
	* Control	AMF	AMF-D	R	R + D
**Phenolics**					
vanillic acid	**10.4**	**14.4**	**2.1**	**27.2**	**17.0**
apigenin 7-glucoside	0.3	1.8	**2.5**	**0.0**	**0.2**
cyanidin3-rytinoside	**0.1**	**0.5**	0.9	**0.0**	0.6
hydroxycaffeic acid	**0.3**	**0.0**	**0.1**	0.9	1.3
isoorientin	**0.5**	0.8	0.9	**0.2**	0.6
isorhoifolin	**0.0**	**0.0**	**0.0**	**0.0**	**0.0**
lariciresinol	0.7	1.6	**2.7**	1.9	**3.4**
luteolin-5-glucoside	**0.5**	0.8	0.9	**0.2**	0.6
quercetin-3-rutinoside	Shoot sp.	Shoot sp.	Shoot sp.	Shoot sp.	Shoot sp.
gamma tocopherol	**20.0**	**123.1**	**5.0**	**6.4**	**42.0**
m-coumaric acid	**0.0**	**0.0**	**0.0**	**0.0**	**0.0**
maachiain	1.0	1.8	**0.5**	1.3	**26.1**
glycinol	1.1	0.9	0.9	1.0	1.0
phaseollin	**94.4**	**14.3**		**10.1**	**149.3**
vestitol	**0.0**	**0.0**	**0.0**	**0.5**	**0.1**
sophorol	0.7	1.5	**0.1**	**0.5**	**4.0**
(6aR,11aR)-3,9-Dihydroxypterocarpan	1.1	**4.5**	0.7	**10.9**	**10.2**
2′-Hydroxyformononetin	1.0	1.8	**0.5**	1.3	**26.1**
astragalin	**0.5**	0.8	0.9	**0.2**	0.6
chalconaringenin	1.1	0.9	0.9	1.0	1.0
delphinidin	**0.3**	1.2	**0.4**	**0.2**	**0.1**
eugenol	**49.0**	0.8	0.8	1.3	1.0
formononetin	**0.2**	1.6	**0.0**	**2.5**	0.9
genistein	**0.3**	0.6	0.9	**2.4**	1.5
genistin	**0.2**	0.6	0.8	**56.9**	**0.2**
homogentistic acid	**10.4**	**14.4**	**2.1**	**27.2**	**17.0**
leucopelargonidin	**4.7**	0.9	1.1	1.1	1.1
lotaustralin	**0.1**	**0.1**	**0.0**	0.6	**0.4**
pelargonidin	**0.3**	0.6	0.9	**2.4**	**0.1**
phenylalcetaldehyde	**0.0**	**0.0**	**0.0**	**0.0**	**0.1**
sinapaldehyde	0.8	1.0	1.0	**2.0**	0.6
vestitone	0.8	0.9	1.0	1.3	1.1
coniferyl acetate	1.1	0.9	1.0	1.1	1.1
astragalin	**0.5**	0.8	0.9	**0.2**	0.6
caffeic acid hexoside	**0.3**	**0.0**	**0.1**	0.9	1.3
coniferaldehyde	**0.0**	**0.0**	**0.0**	**0.0**	**0.0**
diosmetin	0.7	1.5	**0.1**	**0.5**	4.0
eriodictyol	-	-	-	**5.4**	-
**N-containing compounts**					
caffeine	**0.5**	**0.3**	**0.3**	0.8	0.8
cinchonidine	0.9	1.0	**0.3**	**0.1**	1.0
theobromine	0.6	**0.1**	1.2	**2.2**	1.6
portulacaxanthin	**0.5**	**0.1**	**0.1**	**0.1**	1.2
**Terpenes**					
oleanolic acid	1.9	0.6	**0.0**	1.4	1.6
ursolic acid	1.9	0.6	**0.0**	1.4	1.6
menthone	0.6	1.1	0.6	**0.3**	**0.4**
**Sterols**					
desmosterol	1.3	1.4	1.1	1.2	1.0
**Fatty acids**					
myristoleic	0.9	1.5	1.2	**2.0**	**3.9**
octadecanoic acid	0.9	1.6	1.0	1.0	2.3
oleic acid	1.0	1.1	**2.0**	**3.5**	**3.2**
palmitoleic acid	0.9	1.4	1.3	1.9	4.4
pentadecanoic acid	1.3	1.4	1.1	**2.3**	**3.2**
glycerol tributanoate	**0.5**	1.2	**2.8**	1.7	**3.1**

* Control: extracts from non-inoculated plants; AMF: extracts from AMF-inoculated plants (Greek isolate); AMF-D: extracts from AMF-inoculated plants (DAOM strain); R: extracts from *Rhizobium*-inoculated plants; R + D: extracts from *Rhizobium*–AMF double-inoculated plants.

## Data Availability

All data acquired from this research are presented in the main article or the supplementary data.

## Supplementary Materials

The following supporting information can be downloaded at: https://www.mdpi.com/article/10.3390/life14020189/s1, Methodology: Chemical analysis; Figure S1: Anti-inflammatory activities of shoot extracts from *Rhizobium*-inoculated plants; Table S1: List of metabolites identified in the extracts; Table S2: Semi-quantification of selected metabolites.

## References

[1] Jan R., Asaf S., Numan M., Lubna, Kim K.-M. (2021). Plant Secondary Metabolite Biosynthesis and Transcriptional Regulation in Response to Biotic and Abiotic Stress Conditions. Agronomy.

[2] Erb M., Kliebenstein D.J. (2020). Plant Secondary Metabolites as Defenses, Regulators, and Primary Metabolites: The Blurred Functional Trichotomy. Plant Physiol..

[3] Hartmann T. (2007). From Waste Products to Ecochemicals: Fifty Years Research of Plant Secondary Metabolism. Phytochemistry.

[4] Méteignier L.-V., Nützmann H.-W., Papon N., Osbourn A., Courdavault V. (2023). Emerging Mechanistic Insights into the Regulation of Specialized Metabolism in Plants. Nat. Plants.

[5] Smith S.E., Read D., Smith S.E., Read D. (2008). Mycorrhizal Symbiosis.

[6] Begum N., Qin C., Ahanger M.A., Raza S., Khan M.I., Ashraf M., Ahmed N., Zhang L. (2019). Role of Arbuscular Mycorrhizal Fungi in Plant Growth Regulation: Implications in Abiotic Stress Tolerance. Front. Plant Sci..

[7] Zhao Y., Cartabia A., Lalaymia I., Declerck S. (2022). Arbuscular Mycorrhizal Fungi and Production of Secondary Metabolites in Medicinal Plants. Mycorrhiza.

[8] Amani Machiani M., Javanmard A., Habibi Machiani R., Sadeghpour A. (2022). Arbuscular Mycorrhizal Fungi and Changes in Primary and Secondary Metabolites. Plants.

[9] Schweiger R., Müller C. (2015). Leaf Metabolome in Arbuscular Mycorrhizal Symbiosis. Curr. Opin. Plant Biol..

[10] Chaudhary V., Kapoor R., Bhatnagar A.K. (2008). Effectiveness of Two Arbuscular Mycorrhizal Fungi on Concentrations of Essential Oil and Artemisinin in Three Accessions of *Artemisia annua* L.. Appl. Soil. Ecol..

[11] Domokos E., Jakab-Farkas L., Darkó B., Bíró-Janka B., Mara G., Albert C., Balog A. (2018). Increase in *Artemisia annua* Plant Biomass Artemisinin Content and Guaiacol Peroxidase Activity Using the Arbuscular Mycorrhizal Fungus *Rhizophagus irregularis*. Front. Plant Sci..

[12] Domokos E., Bíró-Janka B., Bálint J., Molnár K., Fazakas C., Jakab-Farkas L., Domokos J., Albert C., Mara G., Balog A. (2020). Arbuscular Mycorrhizal Fungus *Rhizophagus irregularis* Influences *Artemisia annua* Plant Parameters and Artemisinin Content under Different Soil Types and Cultivation Methods. Microorganisms.

[13] Sailo G.L., Bagyaraj D.J. (2005). Influence of Different AM-Fungi on the Growth, Nutrition and Forskolin Content of *Coleus forskohlii*. Mycol. Res..

[14] Singh R., Soni S.K., Kalra A. (2013). Synergy between *Glomus fasciculatum* and a Beneficial *Pseudomonas* in Reducing Root Diseases and Improving Yield and Forskolin Content in *Coleus forskohlii* Briq. under Organic Field Conditions. Mycorrhiza.

[15] Bulut M., Wendenburg R., Bitocchi E., Bellucci E., Kroc M., Gioia T., Susek K., Papa R., Fernie A.R., Alseekh S. (2023). A Comprehensive Metabolomics and Lipidomics Atlas for the Legumes Common Bean, Chickpea, Lentil and Lupin. Plant J..

[16] Serventi L., Cai X., Chen R., Dilrukshi N., Su J., Tuange R.P.N., Ham E.E. (2022). Anticancer Properties of Aqueous Extracts from Leguminosae. Nutraceuticals.

[17] Sánchez-Chino X., Jiménez-Martínez C., Dávila-Ortiz G., Álvarez-González I., Madrigal-Bujaidar E. (2015). Nutrient and Nonnutrient Components of Legumes, and Its Chemopreventive Activity: A Review. Nutr. Cancer.

[18] Guang C., Chen J., Sang S., Cheng S. (2014). Biological Functionality of Soyasaponins and Soyasapogenols. J. Agric. Food Chem..

[19] Ranner J.L., Schalk S., Martyniak C., Parniske M., Gutjahr C., Stark T.D., Dawid C. (2023). Primary and Secondary Metabolites in *Lotus japonicus*. J. Agric. Food Chem..

[20] Forslund K., Morant M., Jørgensen B., Olsen C.E., Asamizu E., Sato S., Tabata S., Bak S. (2004). Biosynthesis of the Nitrile Glucosides Rhodiocyanoside A and D and the Cyanogenic Glucosides Lotaustralin and Linamarin in *Lotus japonicus*. Plant Physiol..

[21] Saito S., Motawia M.S., Olsen C.E., Møller B.L., Bak S. (2012). Biosynthesis of Rhodiocyanosides in *Lotus japonicus*: Rhodiocyanoside A Is Synthesized from (Z)-2-Methylbutanaloxime via 2-Methyl-2-Butenenitrile. Phytochemistry.

[22] Tsikou D., Tsiknia M., Nikolaou C.N., Ehaliotis C., Papadopoulou K.K. (2019). The Effect of *Rhizophagus irregularis* and Mesorhizobium Loti Co-Inoculation on *Lotus japonicus*. J. Exp. Mol. Biol..

[23] Hewitt E.J. (1966). Sand and Water Culture Methods Used in the Study of Plant Nutrition. Commonwealth Bureau of Horticulture and Plantation Crops. East Malling, England. Technical Communications.

[24] Hoagland D.R., Arnon D.I. (1950). The Water-Culture Method for Growing Plants without Soil.

[25] Vierheilig H., Coughlan A.P., Wyss U., Piché Y. (1998). Ink and Vinegar, a Simple Staining Technique for Arbuscular-Mycorrhizal Fungi. Appl. Environ. Microbiol..

[26] Mosmann T. (1983). Rapid Colorimetric Assay for Cellular Growth and Survival: Application to Proliferation and Cytotoxicity Assays. J. Immunol. Methods.

[27] Kalousi F.D., Pollastro F., Christodoulou E.C., Karra A.G., Tsialtas I., Georgantopoulos A., Salamone S., Psarra A.-M.G. (2022). Apoptotic, Anti-Inflammatory Activities and Interference with the Glucocorticoid Receptor Signaling of Fractions from *Pistacia lentiscus* L. Var. Chia Leaves. Plants.

[28] Bunim J.J. (1959). A Decade of Anti-Inflammatory Steroids, from Cortisone to Dexamethasone. Summary. Ann. N. Y Acad. Sci..

[29] Liu C.-W., Murray J.D. (2016). The Role of Flavonoids in Nodulation Host-Range Specificity: An Update. Plants.

[30] Tian B., Pei Y., Huang W., Ding J., Siemann E. (2021). Increasing Flavonoid Concentrations in Root Exudates Enhance Associations between Arbuscular Mycorrhizal Fungi and an Invasive Plant. ISME J..

[31] Pei Y., Siemann E., Tian B., Ding J. (2020). Root Flavonoids Are Related to Enhanced AMF Colonization of an Invasive Tree. AoB Plants.

[32] Veitch N.C. (2007). Isoflavonoids of the Leguminosae. Nat. Prod. Rep..

[33] Vitale D.C., Piazza C., Melilli B., Drago F., Salomone S. (2013). Isoflavones: Estrogenic Activity, Biological Effect and Bioavailability. Eur. J. Drug Metab. Pharmacokinet..

[34] Maleki S.J., Crespo J.F., Cabanillas B. (2019). Anti-Inflammatory Effects of Flavonoids. Food Chem..

[35] Elansary H.O., Szopa A., Kubica P., Al-Mana F.A., Mahmoud E.A., Zin El-Abedin T.K.A., Mattar M.A., Ekiert H. (2019). Phenolic Compounds of *Catalpa speciosa*, *Taxus cuspidate*, and *Magnolia acuminata* Have Antioxidant and Anticancer Activity. Molecules.

[36] Kim K.M., Heo D.R., Kim Y.-A., Lee J., Kim N.S., Bang O.-S. (2016). Coniferaldehyde Inhibits LPS-Induced Apoptosis through the PKC α/β II/Nrf-2/HO-1 Dependent Pathway in RAW264.7 Macrophage Cells. Environ. Toxicol. Pharmacol..

[37] Ma Z.-J., Lu L., Yang J.-J., Wang X.-X., Su G., Wang Z.-L., Chen G.-H., Sun H.-M., Wang M.-Y., Yang Y. (2018). Lariciresinol Induces Apoptosis in HepG2 Cells via Mitochondrial-Mediated Apoptosis Pathway. Eur. J. Pharmacol..

[38] Ulanowska M., Olas B. (2021). Biological Properties and Prospects for the Application of Eugenol—A Review. Int. J. Mol. Sci..

[39] Mlala S., Oyedeji A.O., Gondwe M., Oyedeji O.O. (2019). Ursolic Acid and Its Derivatives as Bioactive Agents. Molecules.

[40] Tay K.-C., Tan L.T.-H., Chan C.K., Hong S.L., Chan K.-G., Yap W.H., Pusparajah P., Lee L.-H., Goh B.-H. (2019). Formononetin: A Review of Its Anticancer Potentials and Mechanisms. Front. Pharmacol..

[41] Madadi E., Mazloum-Ravasan S., Yu J.S., Ha J.W., Hamishehkar H., Kim K.H. (2020). Therapeutic Application of Betalains: A Review. Plants.

[42] Jiang L., Wang W., He Q., Wu Y., Lu Z., Sun J., Liu Z., Shao Y., Wang A. (2017). Oleic Acid Induces Apoptosis and Autophagy in the Treatment of Tongue Squamous Cell Carcinomas. Sci. Rep..

[43] Palomino O.M., Giordani V., Chowen J., Alfonso S.F., Goya L. (2022). Physiological Doses of Oleic and Palmitic Acids Protect Human Endothelial Cells from Oxidative Stress. Molecules.

[44] Vandewalle J., Luypaert A., De Bosscher K., Libert C. (2018). Therapeutic Mechanisms of Glucocorticoids. Trends Endocrinol. Metab..

[45] Siddiqui S., Kamal A., Khan F., Jamali K.S., Saify Z.S. (2019). Gallic and Vanillic Acid Suppress Inflammation and Promote Myelination in an In Vitro Mouse Model of Neurodegeneration. Mol. Biol. Rep..

[46] Sozen E., Demirel T., Ozer N.K. (2019). Vitamin E: Regulatory Role in the Cardiovascular System. IUBMB Life.

