# Supplementary material for: Chemical Analysis and Biological Activities of Extracts Isolated from Symbiotic L. japonicus Plants
Source: Life (Basel). 2024 Jan 27;14(2):189. doi: 10.3390/life14020189 (PMC10889931; doi:10.3390/life14020189)
Supplement: Supplementary file 1 [file life-14-00189-s001.zip › Supplementary data - Methodology.pdf]

Chemical Analysis

Chemicals and Reagents

LC-MS grade methanol, water, and formic acid 98–100% were provided by Merck (Darmstadt, Germany). Apigenin 98%, catechin 98%, chlorogenic acid 98%, diosmin 98%, epicatechin 98%, epigallocatechin 98%, hesperidin 98%, isorhamnetin 98%, kaempferol 98%, luteolin 98%, myricetin 98%, myricitrin 98%, naringin 98%, protocatechuic acid 98%, quercetin 98%, quercitrin 98%, rutin 98%, taxifolin 98%, linalool 97%, and elaidic acid 99% were all purchased from Sigma-Aldrich (Stenheim, Germany). Stock standard solutions of all the aforementioned standard compounds were prepared in LC-MS grade methanol at 1000 mg L^{-1} and were afterwards stored in dark brown glass bottles at –20 ^{o}C.

LC-QTOF-MS/MS analysis

Analysis was performed on an ExionAC LC system (SCIEX, MA) that was equipped with two pumps, a solvent degasser, an autosampler, and a controller. The X500R Q-TOF mass spectrometer (SCIEX, Framingham, MA) equipped with an electrospray ionization (ESI) turboVTM source was connected to the LC-system, and it was operated in the negative ionization mode. TOF-MS and TOF-MS/MS data were acquired using a data-dependent acquisition (IDA) electrospray ionization mode. Separation was carried out using a Fortis C18 column (100 mm length, 2.1 mm i.d, 2.6 μm particle size) provided by Fortis (Cheshire, United Kingdom). The temperature of the column was stabilized at 40 °C. The solvents of the mobile phase were: (A) an aqueous solution of 0.1% v/v formic acid and (B) a methanolic solution of 0.1% v/v formic acid. The elution program was gradient, and the flow rate was set at 0.2 mL min $^{-1}$ and 99% (A), gradually dropping to 61% (A) for the next 4 min. The aqueous phase dropped even further to 5% until the 12th min, and it remained stable until the 15th min at a flow rate of 0.4 mL/min. The initial conditions were restored within one min, and for the last four min, the aqueous phase was again at 99% and at a flow rate of 0.2 mL/min to re-equilibrate the column. The QTOF-MS system was equipped with an ESI interface, operating in a negative mode with the following settings: spray voltage of –4500 V, 550 °C heater gas temperature, and 80 V declustering potential. The MS/MS spectra were obtained at a collision energy of 45 V and a collision energy spread of 15 V. External calibration was performed before analysis with a cluster solution provided by SCIEX; additionally, the calibration solution was injected at the beginning of each run for an internal calibration and once per five samples during batch acquisition. Mass spectra were recorded in the range from 50 to 1000 Da at an accumulation time of 0.25 s. MS/MS experiments were conducted using the information-dependent acquisition-dependent mode (IDA) at an accumulation time of 0.08 s for the 10 most abundant precursor ions per full scan. Sample acquisition was monitored by the SCIEX OS software. Extraction ion chromatograms (EICs) were generated using the SCIEX OS software. The established parameters were a mass accuracy window of 5 ppm, a signal to noise threshold of three, a minimum area threshold of 1000, and a minimum intensity threshold of 500.

Screening workflows

Target Screening

A list of 18 target phenolic compounds was created, namely apigenin, catechin, chlorogenic acid, diosmin, epicatechin, epigallocatechin, hesperidin, isorhamnetin, kaempferol, luteolin, myricetin, myricitrin, naringin, protocatechuic acid, quercetin, quercitrin, rutin, and taxifolin [1]. To confirm the identity of the target analytes, the mass accuracies of the precursor ion and the qualifiers, the Rt, and the MS/MS spectra of the real samples and standard solutions were compared.

2.8.2. Suspect Screening

A list of suspect compounds was generated based on the available literature concerning the phytochemical profile of the plant tissues [1]. The mass of the deprotonated ions was calculated based on the molecular formula, and the extracted ion chromatograms were studied using the following parameters: a mass accuracy window of 5 ppm; a signal to noise threshold of three; a minimum area threshold of 1000; and a minimum intensity threshold of 500. The MS/MS fragments were compared to data from SCIEX Natural Products Library for the identification of the unknowns.

Compounds Identification

From the initial target list, apigenin, diosmin, isorhamnetin, kaempferol, luteolin, myricetin, myricitrin, naringin, quercetin, and quercitrin were determined based on their calibration curves to be linear over the range of 0.01–5 mg/L ($r^2 > 0.999$). Each suspect compound was tentatively identified and semi-quantified using the calibration curves of target compounds that have similar structures. Specifically, all the phenolic compounds were semi-quantified using the calibration curve of quercetin, except for vanillic acid, which was semi-quantified using the calibration curve of vanillic acid. Terpenes were semi-quantified with a one-point calibration of linalool, and sterols and fatty acids were semi-quantified using a one-point calibration of elaidic acid.

Calibration Curves

**apigenin**	y = 2E+07x - 4E+07
**diosmin**	y = 3E+06x - 4E+06
**isorhamnetin**	y= 2E+07x - 4E+07
**kaempferol**	y = 2E+07x - 4E+07
**luteolin**	y = 3E+07x + 7E+06
**myricetin**	y = 2E+07x - 265414
**myricitrin**	y = 1E+07x - 502356
**naringin**	y = 8E+06x + 255606
**quercetin**	y = 2E+07x + 2E+06
**quercitrin**	y= 1E+07x + 863092

Reference

1. Ranner, J.L.; Schalk, S.; Martyniak, C.; Parniske, M.; Gutjahr, C.; Stark, T.D.; Dawid, C. Primary and Secondary Metabolites in Lotus Japonicus. *J Agric Food Chem* **2023**, *71*, 11277–11303, doi:10.1021/acs.jafc.3c02709.
