# Supplementary material for: Chemical Analysis and Biological Activities of Extracts Isolated from Symbiotic L. japonicus Plants
Source: Life (Basel). 2024 Jan 27;14(2):189. doi: 10.3390/life14020189 (PMC10889931; doi:10.3390/life14020189)
Supplement: Supplementary file 1 [file life-14-00189-s001.zip › Supplementary Figure S1.pdf]

Supplementary Figure S1

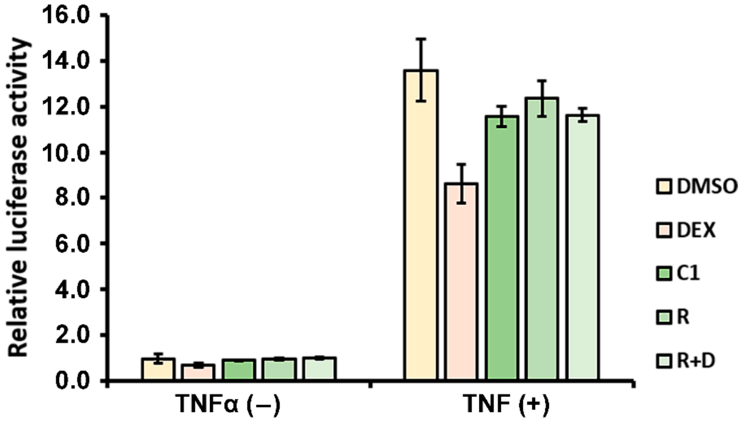

Figure S1. Anti-inflammatory activities of shoot extracts from *Rhizobium*-inoculated plants. Anti-inflammatory activities of extracts from non-inoculated plants grown at 21° C (C1), *Rhizobium*-inoculated (R), and *Rhizobium*-AMF double inoculated plants (R+D) were evaluated by assessment of the suppression of the TNFα-induced NF-κB transcriptional activation in HEK-293 cells. HEK-293 cells were co-transfected with the NF-κB-luciferase reporter and the β-galactosidase reporter constructs and subsequently were treated with 40 μg/ml plant extracts, or with 1 μM DEX, for 6 hours, in the presence or absence of 20 ng/ml TNFα, at hormone-free medium. Results were expressed as relative luciferase activity normalized against β-galactosidase activity. Relative luciferase activity in control TNFα-minus DMSO-treated cells was set at 1. No statistically significant differences were observed by the *Rhizobium*-associated extracts compared to the non-inoculated plant extracts and to the DMSO treatment.
